# From commodity, to customer, to consumer: The Australian beef industry evolution

**DOI:** 10.1093/af/vfy012

**Published:** 2018-06-29

**Authors:** Rod Polkinghorne

**Affiliations:** Birkenwood International Pty Ltd, Murrurundi, New South Wales, Australia

**Keywords:** beef description, Meat Standards Australia beef grading, value-based trading

**Implications**

A consumer focus is essential to align beef industry activity.
Final product description needs to clearly describe a meal experience.
Value-based trading will accelerate profitable industry development.
Industry communication and payment structures must accurately relay value.
Industry culture will dictate the rate of change; the enabling technology exists.
Industry levy funds and prior activity in mandatory animal ID, animal health, and research have laid the foundation for delivery of high-quality branded beef.

## From Commodity to Customer

Cattle were introduced to Australia in 1788, with the first convict settlement from England, as a source of food and to develop self-sufficiency for a new colony. This colonial structure and attitude effectively persisted to the end of the next century with the colony providing increasing supplies of agricultural commodities to England as land was opened up to grazing and a large pastoral industry developed. Production relied on grazing of natural pastures with the climatic extremes producing huge regional and seasonal variation. Early beef exports were tallow and hides due to the distance involved, with the advent of refrigeration leading to the first shipment of frozen beef in 1874. The carcass trade involved a simple commodity; one product to one customer who managed all further distribution. At a local level, cattle trading and processing were also basic with local butchers slaughtering and selling all carcass components to nearby communities. Carcass description was simple and imprecise with Australian export regulations describing carcasses as first, second, or third grade based on dentition, conformation, and fat cover (Polkinghorne and Thompson, 2010).

The introduction of vacuum packaging by the early 1970s became a catalyst for significant change and coincided with other developments of fundamental importance. Critical and diverse factors included a national program to eradicate tuberculosis and brucellosis (the Brucellosis and Tuberculosis Eradication Campaign [**BTEC**]), introduction of Brahman cattle in the north, growth of a cattle feedlot industry, the entry of Britain into the European Union, opening and closing of Japanese market access, increased export of lean trimmings to United States, more demanding domestic food safety regulation, and the rapid growth of supermarkets. Although many of these factors were independent, they both facilitated and demanded industry change. Notably, those who embraced change survived and many previously leading organizations declined or exited the industry.

The growth in export market diversity and centralized supermarket purchasing demanded improved description and imposed new requirements. Animal health and disease status became imperatives to gain or retain market access. BTEC delivered freedom from brucellosis and tuberculosis but, perhaps more significantly, required legislative identification of property of origin and through funding incentives facilitated major infrastructure developments in northern Australia. Higher productivity from tropically adapted cattle combined with herd segregation and more controlled management transformed the pastoral industry from harvesting to a controlled production basis. The feedlot industry complimented grassland production evening out seasonal supply and in many instances delivering a higher quality product. The growth in supermarket sales placed pressure on local butchers and increased central processing. Vacuum packing enabled distribution of individual cuts to multiple markets and in turn encouraged larger integrated slaughter and boning establishments better equipped to manage the complexity of dealing with multiple country import regulations and to develop the required extensive quality assurance systems. While early trading to Japan attempted to maintain “full sets” where all cuts could be marketed on a natural fall basis, inevitably individual customer requirements led to a cut-based trade with each carcass marketed through multiple channels and destinations. The availability of vacuum-packed cuts enabled local butchers to buy selected cuts as boxed beef, reducing the purchase of carcasses. Increasing food safety regulation saw the decline in small localized slaughter and rise of large centralized processing centers. The oil price induced market crash in the early 1970s, coupled with record cattle numbers accelerated consolidation and the evolution of an export dominated industry supplying a large and diverse customer base. New industry organizational structures were introduced whose key objectives were to encourage a consumer focus and collaboration between sectors. A critical factor was agreement to a compulsory levy from all livestock sales to finance research and marketing with government matching the research component. This funding arrangement has been central to driving and facilitating change.

This far more sophisticated industry required better product description, the outcome being the establishment of AUS-MEAT and the AUS-MEAT language in 1987 together with mandatory accreditation for export establishments. Following widespread industry debate, the base AUS-MEAT language delivered carcase classification. The basic mandatory criteria of sex, dentition, P8 (rump) fat depth, and butt (hindquarter muscle) shape described a carcass with common carcass trim reflecting the view that different customers required different specifications and consciously avoiding a grading scheme that assigned quality grades. The language was then augmented by optional chiller assessment measures for marbling, rib eye area, rib fat depth, meat, and fat color that could be used by customers to further define their specification. It was believed that these factors could also be used to underpin quality-grading schemes as promoted by the Australian Lot Feeders Association and other advocates. A critical component of AUS-MEAT implementation was mandatory reporting of all carcass criteria to the producer ensuring that information was communicated regarding carcass suitability and value. Purchasing grids were developed to encourage the production of desired carcass characteristics and facilitated more transparent processor to producer communication.

These developments took the Australian industry to a new level of sophistication which enabled further significant change. Cattle production was more controlled, properties were identified, and carcasses were classified and purchased utilizing common standards and communication enhanced. The processing industry and domestic retail trade were more concentrated and export markets currently include over 57 countries and accounting for over 70% of total production. The substantial export focus forced the industry to be outward looking. It also required compliance with multiple importing company regulations and drove development of very strong industry-wide systems to ensure animals could be traced from birth to slaughter and animal health and treatments validated. The industry was now linked to customers although not necessarily to the ultimate consumer despite them being the sole source of revenue for the industry. A cultural change toward a consumer focus had begun.

## From Customer to Consumer

Despite significant changes in structure, operation, and culture, the beef industry outlook was not encouraging by the late 1980s. As in many large beef-producing countries, beef consumption per capita was falling with consumers reacting to health concerns and alternative protein sources that they judged to deliver superior value. The industry was largely unprofitable with desperate times calling for desperate measures. A defining reaction was the Meat Research Corporation initiating substantial research in the domestic and principal export markets and across the supply chain in each to identify the reasons for declining value. Key findings, common across markets, related to the eating experience; consumers had no confidence in a beef meal experience which varied unacceptably and more so than alternatives. Furthermore, they were confused by the product descriptions and lacked confidence in selection and cooking. The Australian industry reacted by developing a 5-yr strategic plan with principal components centered on research to investigate eating quality variation and potential systems to provide consistent results supported by simple and meal-relevant description.

A core obvious question was to what extent the product varied relative to how much consumer response differed. Consumers were clearly the focus with early research and industry steering group meetings featuring “an empty chair” representing the consumer. Opinions differed widely however as to how to measure consumer response, many believing that untrained consumers would be too erratic to provide useful measurement. On the other hand, objective laboratory and trained panel measurement was found to have a very poor relationship to consumer data. After considerable initial research, very strict protocols for sensory testing using untrained consumers covering all facets of product collection, sample fabrication, cooking, and serving were developed to ensure that any scoring difference could be attributed to either the consumer or the beef. To counteract individual consumer variation, it was determined that utilizing10 consumers with clipping procedures to manage outliers provided a reliable and repeatable measure. A composite eating quality (MQ4 score) statistic was developed from the data to represent the appropriate weighting of tenderness, juiciness, flavor, and overall satisfaction. This was adopted as the measure for all subsequent research and agreed to be a dynamic standard that should be reviewed and if necessary adjusted in response to any change in consumer response over time. Further statistical analysis established appropriate cutoff scores to separate product deemed to be unsatisfactory, good everyday, better than everyday, or premium quality. Consumer sensory testing was then initiated on a large scale and used as the primary measure to quantify product variation and relationships to multiple factors including the source animal, processing procedures, product ageing, and cooking styles. More detail of the sensory protocol development is reported by Watson et al. (2008a).

This approach, together with the aggregation of data from all trials, has been of fundamental importance to subsequent development. Consumers were found, in aggregation, to be remarkably consistent with little demographic influence and consistently able to identify small differences in eating quality; in fact of superior sensitivity to the common laboratory measures.

An early outcome was proof that the product did vary and varied widely. The industry had a serious problem. This was not entirely unexpected given the huge variation in Australian production systems ranging from tropical grassland to cold climate improved pastures, from *Bos indicus* to British and European cattle and crosses, between grass and feedlot production systems with further extremes in age, weight, and finish. This created significant challenges to develop a system that could adequately predict consistent consumer outcomes across such a diverse base.

Initial work, following research reported by Morgan (1992) and others, attempted to develop production pathways utilizing a palatability critical control point approach where product could be adequately segregated by meeting or exceeding a number of criteria such as maximum “*Bos indicus* percentage”, maximum dentition, minimum rib fat and marbling, carcass weight relative to ossification coupled with requirements for handling and transport pre slaughter, electrical carcass stimulation, pH and temperature decline, and minimum cut aging. Consumer score standards were agreed and multiple criteria combinations tested across cattle groups utilizing a grilled striploin for assessment. Several successful pathways were established enabling early industry trial. Of concern however was the lack of specificity. If standards were set sufficiently high to guarantee consumer satisfaction, a large proportion of satisfactory product was rejected due to failing to pass one of the criteria but offset by exceeding others. Consequently, there was a commercial conflict between acceptable consumer outcomes and acceptable commercial volume.

**Figure 1.**
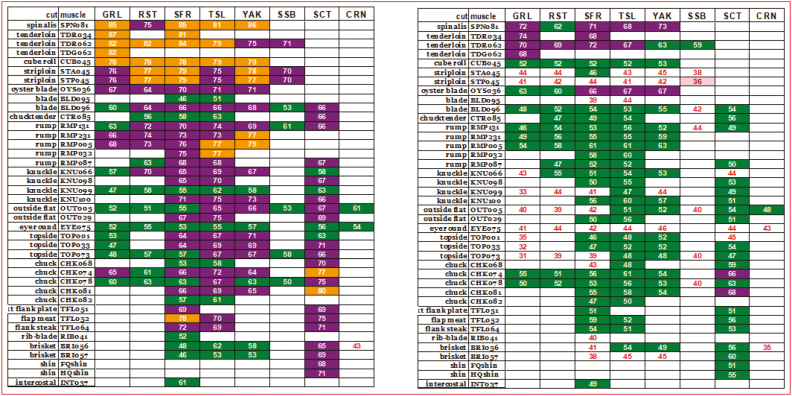
Example output (MQ4 scores) for muscle and cook combinations for a higher and lower grade carcass.

The solution proved to be an interactive statistical model that calculated the direct and interactive effect of all criteria to produce an aggregate total score utilized to segregate to the appropriate quality band or grade. With this approach, commercial application became feasible as the majority of acceptable product was identified and rejected product was largely accurately predicted. Testing was widened to evaluate multiple cooking methods and an expanded range of muscles. This testing generated further challenges as alternative cooking methods often produced widely different scores from the same muscle. It was obvious that a prediction model needed to be muscle and cooking method specific. It was expected, and confirmed, that different carcass muscles or cuts would have different eating quality ratings, but assumed that the relationship between muscles may be constant. This was not the case. While within a carcass a relative ranking could be calculated, the relativity differed within each carcass making a single carcass grade inappropriate. As further data were accumulated, it was established that most grading input factors impacted differently on individual muscles. Post mortem aging had virtually no effect on tenderloin (*M. psoas major*) but a large impact on striploin (*M. longissimus dorsi*), marbling differed significantly between muscles as did *B. indicus* influence, hormonal growth implant use and weight for age factors (Polkinghorne, 2005). These individual relationships could be accommodated within a prediction model, with resulting outputs comprising a matrix of individual muscle × cook outcomes for each individual carcass further adjusted for days aged post mortem. This prediction model approach was adopted with the model upgraded progressively as new data were evaluated (Watson et al., 2008b). Example output for higher and lower grade carcass is displayed in Figure 1. The predicted MQ4 scores for each muscle by cook combination are shown within the matrix with the background color related to 5* (gold), 4* (purple), 3* (green), or fail (no color) categories. As shown, there is a considerable MQ4 difference for common muscles between the two carcasses and a wide distribution of quality within each carcass. This illustrates the inherent conflict between clear consumer-focused communication, single grades for entire carcasses and retail description by cut.

The Meat Standards Australia (**MSA**) system moved from the initial pathways base to a prediction model form in 2000. Additional MSA criteria used as prediction inputs were added to the AUS-MEAT language and to mandatory producer reporting. Further discussion of the evolution of MSA is provided by Polkinghorne et al. (2008a).

The value of the feedback data to the producer was enhanced by industry adoption of mandatory lifetime electronic individual animal identification. This enabled herd management systems to be linked electronically to carcass and grading data at individual animal level for analysis and improvement. Across industry cooperation in animal traceability, implant status and declarations regarding feed regimes and animal health treatments have provided a strong export capability that is able to meet the demands and desires of global customers. The processing sector has developed very strong quality assurance and exemplary hygiene practices necessary to achieve long chilled product shelf life and essential for long distance sea freight.

The individual muscle × cook output provided a highly relevant consumer-focused base able to describe individual meal satisfaction from a wide range of carcasses. This new capability created an opportunity to reappraise retail product description and address challenges relating to carcass and cut identification, sorting during boning and pricing, and reporting to suppliers. Although traditionally cut was the primary retail description, the growing MSA data cast doubt on its relevance as there was extreme eating quality variation within each cut and for each cooking method. Within grilled cuts, MQ4 variation was typically close to 70 points on a 100-point scale with roasts and slow-cooked muscles having a 50 or greater range. Given these ranges, it could be argued that cut description might in fact be more confusing than helpful to the consumer, further supported by reports that consumers find cut names confusing. Also of note was that for the 40 muscles predicted in each carcass many fell within a common grade within cooking method. This further challenged cut-based description; if cut did not provide an indication of eating quality and multiple cuts from a carcass had equivalent performance, why not describe by cooked outcome—4* grill or 3* roast? An early commercial evaluation of retailing based on cooked result rather than cut was reported by Polkinghorne et al. (2008b). This study also reported considerable range in individual carcass value based on differences in eating quality and yield.

Industry systems were developed to sort carcasses based on setting minimum eating quality MQ4 levels for selected cuts enabling segregation into boning runs. Industry uptake progressed from around 300,000 head in 1999/2000 to over 2.8 million in 2016/2017 representing 46% of the annual kill. The system is voluntary with growth driven by commercial response. Premiums to producers over nongraded product have been estimated at $153 million (Meat Standards Australia, 2017) returned to the farm gate in 2015/2016 and improved returns across all sectors. These results are interpreted as an increase in consumer value and are most encouraging. The industry has moved substantially toward a consumer focus with more consistent product from grouping within eating quality bands. This has provided a solid platform for company branding where the consistent eating quality base guarantees consumer satisfaction and further value and differentiation can be leveraged through provenance and emotional positioning. Over 150 MSA-based brands are currently registered and underpinned by MSA grading. As reported by Grunert et al. (2004), it is critical that the actual consumer outcome matches or exceeds the expectation at purchase. Provenance and emotional branding relating to animal welfare, feeding systems, natural product, and organics can provide strong purchase intent. If the product fails to deliver however, the message is lost, whereas as an outstanding eating experience builds faith in the brand and its related attribute claims.

## The Potential

Vast potential still remains to transition the Australian industry further toward a consumer focus and to increase efficiency and profitability through aligning reporting and payment with consumer value. Further differentiation into more defined eating quality categories and greater utilization and sale of secondary cuts based on consumer outcomes can add substantial value and continue a transition toward a fully consumer-focused industry. The issues discussed, while related to an Australian experience, are believed to be equally relevant on a broad global basis. Beef has become a premium product priced substantially above competing proteins. This can only continue if consumers find value in the offer which critically requires consistent satisfaction and confidence in supporting industry systems including food safety, environmental, and welfare credibility.

By nature, the beef industry is global, as are consumer responses and their concerns, with allowances for some regional variation or emphasis on particular issues. As shown with past crises such as BSE or *E. coli* O157:H7 contamination, a falling tide lowers all ships with immediate and universal fall in demand across both affected and unaffected suppliers. It is contended that variable or poor eating quality experiences exert equal or greater impact, with the difference being they are less visible and measured over a longer period reflecting the consumer’s value judgment through reduced volume or prices. Conversely, improved and consistent eating experiences can grow demand.

**Figure 2.**
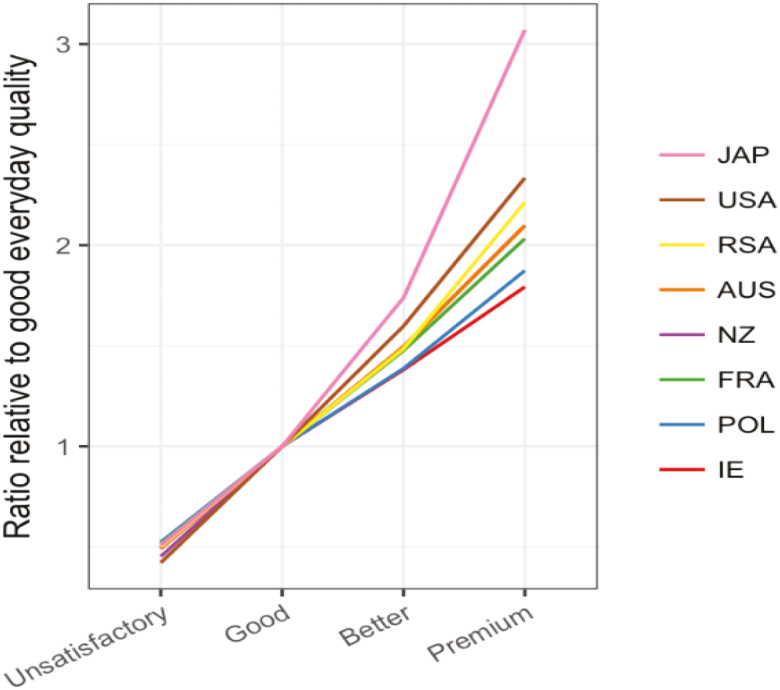
Willingness to pay ratios by country.

Willingness to pay data collected in conjunction with consumer testing in several countries, as depicted in Figure 2, emphasizes this assertion with unsatisfactory product rated as half the value of good everyday quality with better than every day rated around 1.5 times and premium quality 1.8 to 3 times the nominated everyday price.

Consumer response is similar across regions and well-illustrated by work under common protocols in South Korea (Thompson et al., 2008), Northern Ireland (Farmer et al., 2010), Japan (Polkinghorne et al., 2014), France (Legrand et al., 2013), South Africa (Thompson et al., 2010, United States (Polkinghorne 2007), and Poland (Polkinghorne, unpublished data). This has been recognized by the groups involved and stimulated the formation of a research foundation to facilitate the publication of common consumer testing and carcass-grading input protocols under the United Nations Economic Commission for Europe (**UNECE**) and to enable data sharing and collaboration. Given the global nature of the beef trade, there is considerable mutual benefit in a shared knowledge of consumer populations and in reliably identified consistent quality product that can result from the implementation of consumer-based grading.

**Figure 3.**
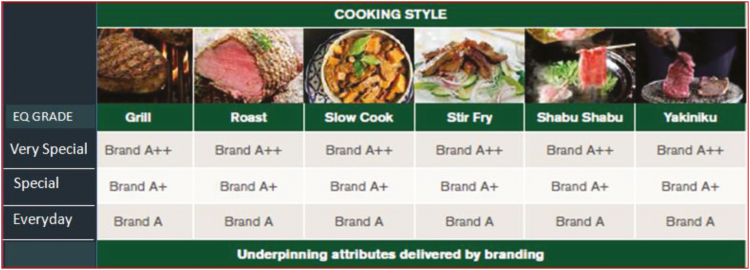
Conceptual consumer-oriented retail description.

While the logic of moving from cut-based description to a consumer-focused outcome is strong, tradition and caution have constrained broad adoption. Figure 3 displays a conceptual matrix that might be translated to a retail cabinet.

Under this concept, purchasing choice is simplified to a decision on meal type for a specific occasion with branding delivering the alternative quality levels with potential brand-provenance rounding out a choice between clear value propositions. The enabling technology and detail of delivering a simple choice are retained by industry as it should be.

This concept is also applicable, and ripe for adoption, in the ready-to-eat meal category where a cooked or ready-to-heat meal is described by meal rather than ingredient. A significant industry opportunity, and challenge to the status quo, is to extend eating quality categorization to traditional secondary cuts, to segregate these cuts into consistent defined quality levels and to raise the inherent eating quality of unsatisfactory product by value adding processes.

Consumer value is inherently linked to meal satisfaction relative to price, with some potential addition for believable provenance claims. The consumer value derived from a carcass is consequently in effect the sum of the meals produced extended by suitable pricing per unit related to the quality band. To the packer, the value is adjusted further by yield, representing the weight of meals produced per kg of carcass weight, and by-product sales. As reported by Polkinghorne et al. (2008b), cattle consignments invariably include a range of value after adjustment to common weight. This range is extreme and typically several hundred dollars per head at the farm gate. If the industry can identify and multiply the higher-than-average cattle and replace the poorer end, a significant latent pool of revenue can be directed to the consumer, retailer, packer, or livestock supplier with direct impact on industry profitability. As eloquently stated by Cross and Savell (1992) “without market differentiation, no real incentives are given for producers to purchase “better” breeding stock, for feeders to sort animals to better meet slaughter endpoints or not to overfeed, for packers to trim boxed beef more closely rather than selling excess fat down the chain and for retailers and purveyors to purchase products differently from in the past”. Sixteen years later, the statement remains pertinent, but perhaps more actionable now as new technologies for accurate carcass yield measurement, sophisticated technology for identifying and sorting individual cuts, accurate consumer-based grading at a meal level and advanced electronic identification and communication technology to collect, collate and transfer information are available.

Adoption of transparent value-based trading systems can drive massive positive industry development and deliver a consumer value focus through payment at all supply-chain transaction points with attendant profitability improvement. The restraining factor is no longer technology or capability but rather industry culture and caution. Concerted action by the global industry can radically improve the consumer value of beef through improved product consistency and clear meal level description. The future is in our hands!

## About the Author

**Dr Rod Polkinghorne**, OAM, developed the base concept and managed all subsequent scientific development of Meat Standards Australia (MSA) as original team leader. He continues to manage ground-breaking research relating to MSA and has been recognized with awards such as an Order of Australia medal for services to the beef industry and Honorary Doctorate from Murdoch University. He is a pioneer of the Australian feedlot industry, a leading innovator in the Australian dairy industry with marketing of specialty ultra-filtered milk and intensive production and a leader of consumer-based beef grading research for over 20 years. He is a prolific author, an Adjunct Professor of Meat Science at the University of New England and has led collaborative consumer research projects for red meat in Japan, Korea, United States, Northern Ireland, Ireland, France, South Africa, and New Zealand. From his own herd Rod developed a paddock to plate branded beef production and retailing business with deboning, retail cut fabrication, cooked meal production, retail, and IT systems components. Rod developed operating systems to utilize MSA eating quality estimates combined with yield parameters to optimize revenue from each beef cut in every animal.
